# Real-Time Monitoring of Non-linear Suicidal Dynamics: Methodology and a Demonstrative Case Report

**DOI:** 10.3389/fpsyg.2016.00130

**Published:** 2016-02-15

**Authors:** Clemens Fartacek, Günter Schiepek, Sabine Kunrath, Reinhold Fartacek, Martin Plöderl

**Affiliations:** ^1^Suicide Prevention Research Program, Paracelsus Medical UniversitySalzburg, Austria; ^2^Department of Suicide Prevention, University Clinic of Psychiatry and Psychotherapy, Christian Doppler ClinicSalzburg, Austria; ^3^Department of Clinical Psychology, Christian Doppler ClinicSalzburg, Austria; ^4^Institute of Synergetics and Psychotherapy Research, Paracelsus Medical UniversitySalzburg, Austria

**Keywords:** suicide prevention, real-time monitoring, non-linear dynamic systems, risk assessment, early warning system, critical instability

## Abstract

In recent years, a number of different authors have stressed the usefulness of non-linear dynamic systems approach in suicide research and suicide prevention. This approach applies specific methods of time series analysis and, consequently, it requires a continuous and fine-meshed assessment of the processes under consideration. The technical means for this kind of process assessment and process analysis are now available. This paper outlines how suicidal dynamics can be monitored in high-risk patients by an Internet-based application for continuous self-assessment with integrated tools of non-linear time series analysis: the Synergetic Navigation System. This procedure is illustrated by data from a patient who attempted suicide at the end of a 90-day monitoring period. Additionally, future research topics and clinical applications of a non-linear dynamic systems approach in suicidology are discussed.

## Introduction

Modeling psychopathological processes with non-linear dynamic systems has been suggested by numerous scholars (Schiepek and Tschacher, 1992; Barton, 1994; Belair et al., 1995; Ehlers, 1995; Heiby, 1995; Breakspear, 2006; Tschacher and Junghan, 2009; Haken and Schiepek, 2010; Bystritsky et al., 2012; Yang and Tsai, 2013). Non-linear dynamic systems are also an emerging field in suicidology (Mishara, 1996; Ramsay, 1997; Rogers, 2003; Large, 2010; Rogers and Lester, 2010; Schiepek et al., 2011b; Sawhney, 2012), contributing new perspectives on the emergence and prediction of suicide-related phenomena. Before discussing this any further, we introduce some basic concepts about non-linear dynamic systems (for a detailed description see Stam, 2005; Strunk and Schiepek, 2006).

Non-linear dynamic systems are referred to as dynamic because they exist over time and can realize processes (an der Heiden, 1999), i.e., a dynamic system determines the evolution of a system over time (e.g., trajectories of suicide ideation) given an initial state (Stam, 2005). Dynamic systems are non-linear when their input and output are not proportional to each other (Stam, 2005). A suicidological example would be that marginal stressors (input) can trigger suicidal behavior (output) or that substantial stress does not lead to suicidal behavior (Schiepek et al., 2011b). Moreover, non-linear dynamic systems are defined by four important characteristics (Strunk and Schiepek, 2006): first, they consist of at least one variable in the case of discrete processes or of at least three variables in continuous processes. Second, there is at least one non-linear relationship between variables. Third, the interactions between variables combine positive (activating) and negative (inhibitory) feedback loops. Fourth, the system is dissipative, i.e., thermodynamically open, energy consuming and energy exchanging with the environment, and driven by so-called control parameters.

Non-linear dynamic systems potentially produce a wide range of dynamic patterns referred to as attractors since they “attract” trajectories (sequences of system states) from within a particular catchment area (basin) and restrict the system dynamics to their pattern. Deviating input or system states out of this pattern are damped away. The prediction horizon of non-linear dynamic systems is limited (Schiepek et al., 2011b): some types of attractors (e.g., point attractors, limit cycles, tori) enable long-term predictions if their parameters and boundary conditions remain stable or controllable. However, in living systems this cannot be taken for granted (Schiepek et al., 2015). Other types of attractors are more complex and refer to behaviors that can’t be predicted in the long run, even if the generating system operates completely deterministically and is known in detail (Strunk and Schiepek, 2014). The most prominent type of complex dynamics is deterministic chaos, which became familiar as the butterfly effect in the 1960s through meteorologist Lorenz (1963). Chaotic attractors are complex behaviors between regularity and noise that can’t be predicted on a long-term basis because even smallest differences in initial conditions (dynamic noise) would lead to a massive divergence of the trajectories over time (Ott, 2002; Schuster and Just, 2006; Salvatore and Tschacher, 2012). So-called phase transitions are another characteristic of non-linear dynamic systems, with reference to transitions from one attractor to another. During phase transitions, systems are in an instable symmetry state where even minimal input from the outside or internal random fluctuations can be crucial for the system’s next emerging attractor. Hence, non-linear dynamic systems in a phase transition are at the mercy of small fluctuations; they are highly unstable and not predictable (Strunk and Schiepek, 2006).

Empirical evidence supports some of the predictions made by the theory of non-linear dynamic systems in psychiatry and psychotherapy. Studies reported chaotic properties in psychopathological processes (Gottschalk et al., 1995; Tschacher et al., 1997), or the emergence of critical instabilities at significant changes (phase transitions) in psychopathology and psychotherapy (Schiepek et al., 2003; Aderka et al., 2012; Gumz et al., 2012; Schiepek et al., 2013, 2014). Both are considered as specific characteristics of non-linear dynamic systems (Strunk and Schiepek, 2006, 2014).

Several suicidologists have suggested modeling suicidal processes within the framework of non-linear dynamic systems (Mishara, 1996; Rogers, 2003; Hjelmeland and Knizek, 2010; Large, 2010; Rogers and Lester, 2010; Schiepek et al., 2011b; Sawhney, 2012; Fartacek et al., 2015). From this perspective, the failure of long-term predictions of suicidal behavior (Large and Ryan, 2014) is not a consequence of unspecific or unknown risk factors but a consequence of the inherent complexity of the underlying system, related to chaos, phase transitions, or unstable dynamics and boundary conditions (Strunk and Schiepek, 2006). Furthermore, the familiar clinical experience that marginal stressors can lead to suicidal crises or, inversely, that substantial stress does not cause increased suicide risk, is in line with the tenets of non-linear dynamic systems: the impact of external input depends by far more on the degree of stability or instability of a system than on the input itself. Moreover, from a non-linear dynamic systems perspective, suicidal states can be viewed as specific attractors of mental functioning – “the suicidal mode” – characterized by severe restrictions of cognitive, emotional, and behavioral adaptivity (Rudd, 2000).

Until now, non-linear dynamic systems theory has been applied to suicidology only metaphorically (Rogers, 2003). Empirical studies that investigate specific dynamic properties of non-linear dynamic systems in suicidal processes (e.g., chaos, phase transitions) are still missing, likely due to a lack of necessary research tools, which have become available only recently. These tools must provide continuous and frequent assessments of suicidal processes with equidistant sampling rates (time-sampling) and provide algorithms applicable to short, coarse-grained, and non-stationary time series that enable the identification of typical dynamic properties of non-linear dynamic systems. Time-sampling procedures can be realized using electronic diaries such as the *experience sampling method* (Csikszentmihalyi and Larson, 1987), *ecological momentary assessment* (Shiffman et al., 2008), or the *Synergetic Navigation System* (SNS; Schiepek, 2003; Schiepek et al., 2015). These methods have already been used in suicidological applications (Nock et al., 2009; Ben-Zeev et al., 2012; Palmier-Claus et al., 2012, 2013; Thompson et al., 2014), but not from a non-linear dynamic systems background. For ethical reasons it is important to note that the few available studies do not hint at a potential iatrogenic effects of high-frequency monitoring of suicidality (Clum and Curtin, 1993; Husky et al., 2014; Law et al., 2015). However, only the study of Law et al. (2015) was a randomized controlled trial and thus provided an appropriate study design; therefore, more studies are needed to find out if a negative side-effect of monitoring can be ruled out.

The identification of dynamic properties of such non-linear dynamic systems in process data is based on non-linear time series analysis. Classical algorithms, such as the estimation of fractal dimensionality, require very long, highly resolved, and stationary time series data (Grassberger and Procaccia, 1983a,b). However, stationarity, i.e., invariant statistical characteristics across time (Molenaar and Campbell, 2009), cannot be assumed in human systems. Furthermore, the compliance needed for assessing thousands of measures is unrealistic even in highly motivated patients. This highlights the need for alternative algorithms applicable to short, coarse-grained and non-stationary time series. Schiepek et al. (2015) suggested algorithms which can be implemented in time series from at least 30 measurement points, such as dynamic complexity (Schiepek and Strunk, 2010), permutation entropy (Bandt and Pompe, 2002), recurrence plots (Webber and Zbilut, 1994), or dynamic synchronization matrices (Haken and Schiepek, 2010). These procedures are available in the SNS (Schiepek, 2003; Schiepek et al., 2015; see Materials and Methods).

The aims of this paper are twofold. First, we would like to demonstrate the assessment and analysis of the suicidal process in clinical practice using the SNS and some of the algorithms mentioned above. To make the demonstration easier to follow, we have used the data from a high-risk patient. This way we can discuss how the SNS can be used both as a research as well as a clinical tool. Second, we outline future research directions and clinical applications.

## Materials and Methods

### The Patient

In the following description some personal information has been altered to ensure anonymity. The patient was a 54-year-old married female with one child. The patient had been absent from work for several months after a previous inpatient treatment. She was admitted to our department because of another depressive episode, likely resulting from not being able to work and feelings of isolation at home. She was diagnosed with recurrent depressive episodes and problematic alcohol use during extended periods of her life, but not presently. Different symptoms of a Cluster B personality disorder below the clinical threshold were identified. For several years the patient had been continuously treated in psychiatric and psychotherapeutic outpatient settings. Biographically, the patient reported some stressful life events, such as early separation from her mother, violent verbal and physical assaults in a dormitory, and a traumatic therapeutic relationship during her first outpatient psychotherapy where she felt absolutely dependent on her therapist. At the time of monitoring she suffered from isolation and depression, was incapable of working, and did not see any meaning in life anymore. She had already made several suicide attempts, including nearly lethal ones, e.g., by jumping in front of a truck during previous inpatient treatment and by overdosing on alcohol and prescription drugs. There were also several aborted suicide attempts where the patient was very close to jumping from dangerous heights.

The current inpatient treatment at the suicide prevention department lasted several weeks. Monitoring with the SNS started on the 19th day of her hospital stay, continued for 91 days, and stopped abruptly after a suicide attempt by overdosing on benzodiazepines. Subsequently, the patient was treated in the closed ward of the hospital for several days which made it impossible to continue the self-assessment by the SNS. The exceptional occurrence of a suicide attempt during a period of monitoring was the reason for selecting this patient for this case report.

### The Synergetic Navigation System

The SNS is a web-based instrument that was developed for real-time monitoring of human change processes (e.g., psychotherapy, counseling, Internet-based coaching, suicide prevention). The sampling rate can be arbitrarily defined. Following good practice in feedback-informed psychotherapy, a sampling rate of once per day was used (Schiepek et al., 2015). Technically, assessments can be done from a third person’s perspective (e.g., by relatives, clinicians, or therapists) or by patients themselves. In this case, patients’ self-reports by the *Salzburg Suicide Process Questionnaire* (see below) were used. The items of this Questionnaire combine Likert-type scales and visual analog scales. The resulting data are visualized by time series graphs for all items, which can be analyzed by algorithms such as dynamic complexity, recurrence plots, permutation entropy, or item-to-item intercorrelation matrices. The parameters are calculated within a moving window of arbitrary width (in our case, 7 days). In the following description we concentrated on dynamic complexity, synchronization patterns, and recurrence plots.

#### Dynamic Complexity

**FIGURE 1:**
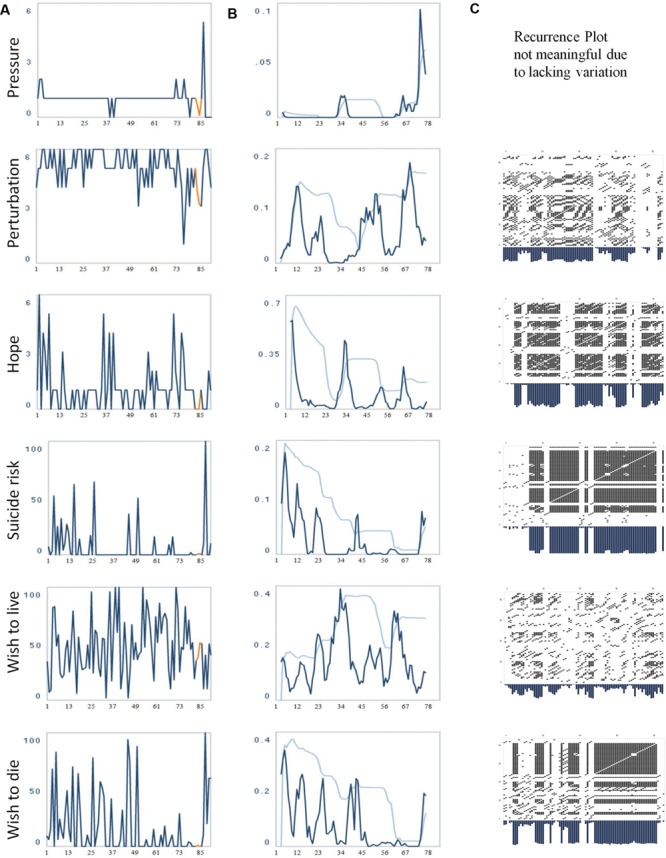
**(A)** Evolution of the items “pressure,” “perturbation,” “hope,” “suicide risk,” “wish to live,” and “wish to die.” The numbers on the *x*-axis correspond to days of monitoring. **(B)** The evolution of the dynamic complexity corresponding to these items (calculated in a moving window of 7 data points [=days]). The two additional curves (light blue) represent a 95 and 99% confidence interval, calculation based on 21 data points. The numbers on the *x*-axis correspond to days of monitoring. **(C)** Recurrence Plots of the time series. Each of the items was embedded into a three-dimensional phase space with delay 1 (t-1, t-2, t-3). Radius: 20 (item 10/Perturbation), 18 (item 11/Hope), 13 (item 14/Suicide risk), 25 (item 15/Wish to live), 25 (item 16/Wish to die). Periods of instability or transients have reduced recurrence, i.e., very few dots, and correspond to increased dynamic complexity (middle column), and vice versa, periods with distinct recurrent signature correspond to a restricted dynamic complexity.

**FIGURE 2:**
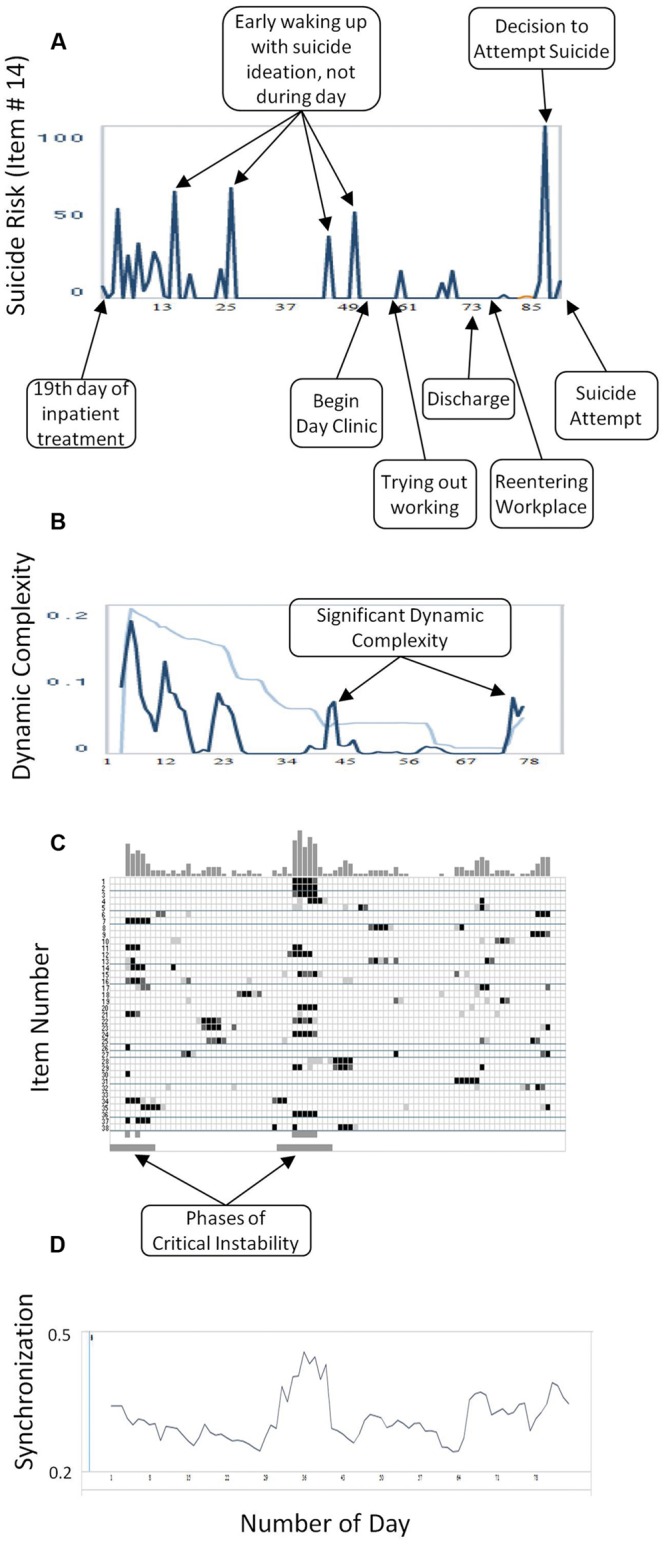
**(A)** Critical Incidents: the numbers on the *x*-axis correspond to days of monitoring. **(B)** Dynamic Complexity of item # 14 (blue line) and the confidence interval (light blue line). Increases of dynamic complexity indicate periods of critical instabilities in a change process. The dynamic complexity is calculated in a gliding window with window width of 7 points. The numbers on the x-axis correspond to days of monitoring. **(C)** Complexity Resonance Diagram: each row represents one item of the Salzburg Suicide Process Questionnaire. Light gray, dark gray, or black dots indicate three significance levels of the dynamic complexity (*p* < 0.050, *p* < 0.025, *p* < 0.010, respectively). The Complexity Resonance Diagram indicates periods of significantly increased complexities getting in resonance to each other, and by this, appear at the same time (vertical structures). Usually, those periods can be seen as the critical instabilities of a process. In this Complexity Resonance Diagram the two critical periods of the 3 months of monitoring are marked below. The top histogram summarizes dynamic complexities across items. **(D)** Synchronization: degree of intercorrelation between items. The numbers on the *x*-axis correspond to days of monitoring.

The dynamic complexity algorithm (Schiepek and Strunk, 2010) combines a fluctuation measure *F* and a distribution measure *D* in a multiplicative way. The algorithm was designed to identify critical instabilities preceding phase transitions (Haken, 2004; Schiepek et al., 2014). Dynamic complexity is applicable to short, coarse-grained, interval-scaled, and equidistantly time-sampled real-world data without any further assumptions (e.g., concerning distribution characteristics, scale resolution, or length of time series). The fluctuation *F* is sensitive to the amplitude and frequency of changes in a time signal (volatility), and the distribution *D* scans the scattering of values realized within the range of possible values (for a detailed description, see Schiepek and Strunk, 2010; for clinical applications, see Schiepek et al., 2003, 2014; Gumz et al., 2012). The dynamic complexity is usually calculated within a data window of seven measurement points, moving from day to day over the whole time series. This procedure results in a time series of the dynamic complexity of each item. To identify statistical significance of the current dynamic complexity, a dynamic 95 and 99% confidence interval is applied, which is based on the distribution of the last 21 complexity values (see **Figure 1**). In addition, the evolving dynamic complexity of each item can be visualized by the *Complexity Resonance Diagram* (see **Figure 2**). Each line of the Complexity Resonance Diagram represents one item. The Complexity Resonance Diagram highlights simultaneously increased dynamic complexities across items, indicating periods of critical instability of the system. Instead of an analogous visualization of the dynamic complexity, the Complexity Resonance Diagram visualizes different significance levels of the complexity values. The significance refers to the variability within the respective complexity course (auto-calibration) and is marked at three levels: light gray (*p* < 0.050), dark gray (*p* < 0.025), and black (*p* < 0.010).

#### Synchronization Patterns

The absolute (sign-independent) values of all item-to-item correlations of a questionnaire are averaged within a moving window and are presented by a time series. This graph is a measure of the time-dependent overall synchronization strength of all items of a questionnaire or of all subsystems of a system. It indicates the degree of synchronization of the subsystems or elements which corresponds to the “enslaving” power of a global attractor (Haken, 2004). In addition to this overall measure, the inter-item correlations can be presented in a triangular correlation matrix of all n(n-1)/2 correlation coefficients (n is the number of items) color-coded from –1 [dark red] to 0 [white] to +1 [dark green]. The sequence of correlation matrices is calculated within a moving window (the window width is left up to free choice; standard: 7 measurement points). In the SNS, a marker can be dragged along the overall synchronization to display the sequence of synchronization patterns movie-like over time.

#### Recurrence Plots

This method identifies recurrent patterns within a time series by a time × time diagram (Eckmann et al., 1987;Webber and Zbilut, 1994). The time series is embedded in a phase space which is constituted by time-delay coordinates, with the time-delay defined by the first minimum or zero crossing of the autocorrelation function of the time series. Each vector (point) in the phase space corresponds to a segment of the time series. The number of elements (values) of the vector corresponds to the number of time-delay coordinates, and the distance between the values is defined by the time-delay. For example: if the time-delay corresponds to 1, and the phase space has 3 coordinates, the first vector represents the 1st, 2nd, and 3rd value of the time series; the second vector the 2nd, 3rd, and 4th value, and so on, until the time series is completely embedded. Vector points, which are placed at small Euclidian distances in the phase space (neighbors), represent similar value sequences (patterns) occurring within the time series. According to a selected threshold (radius of a hyper-cycle), neighbors which represent similar (recurring) patterns are marked by a dot in the time × time plot (**Figure 1**). By this procedure, patterns of similar (recurrent) dynamics and non-recurrent dynamics (transients, e.g., periods of critical instability) are made visible. This method allows for a differentiation of recurrent patterns behaviors (e.g., cyclic processes) from complex behavior (e.g., phase transitions), visible as empty stripes in the recurrence plot. Recurrence plots and Complexity Resonance Diagram should be complementary in as far as periods without recurrent dynamics (transients) correspond to increased dynamic complexity (e.g., periods of critical instabilities), and conversely, periods with distinct recurrent signatures often correspond to a restricted dynamic complexity.

### The Salzburg Suicide Process Questionnaire

The Salzburg Suicide Process Questionnaire comprises 38 items that were either composed by the authors or taken from established questionnaires. Items drawn from other questionnaires were selected by the criterion of the best item-scale correlation. Some of the items had been tested in other contexts previously. Most of the items had to be translated into German. If necessary, the items were rephrased with “today” to let the participant focus on the present day. To avoid inducing negative moods by the daily assessments, we used a nearly balanced selection of items with positive and negative emotional valence. Since the sample of cases is still small, psychometric data of the Salzburg Suicide Process Questionnaire have not been reported until now. **Table 1** provides an overview of the items.

**Table 1:** Items of the Salzburg Suicide Process Questionnaire.

No.	Item	Response	Factor	Source and Comments
1	^∗^Today I was convinced that it is good to be alive on earth.	Likert	Basic worthiness of one’s existence	Item # 39 from the Test zur Existentiellen Motivation (TEM), Eckhardt (2001)
2	^∗^Today I felt that “basically it’s good that I exist.”	Likert	Basic worthiness of one’s existence	Item # 49 from the TEM
3	^∗^Today I thought that I contribute to the well-being of my family/friends.	Likert	Burdensomeness/Connectedness	Item # 5 from the Interpersonal Needs Questionnaire (INQ), Van Orden et al. (2008), but targeted at family/friends. This item correlated strongest with the burdensomeness scale (Van Orden, personal communication, April 12, 2009)
4	^∗^Today I thought that I contribute to the well-being of people in my current environment.	Likert	Burdensomeness/Connectedness	Item # 5 from the INQ, but targeted at the people in the current surroundings (e.g., clinic)
5	Today I felt disconnected from other people.	Likert	Burdensomeness/Connectedness	Item # 14 from the INQ. This item correlated strongest with the connectedness scale (Van Orden, personal communication, April 12, 2009)
6	The thought of taking my life made today’s situation more bearable.	Likert	Chronic Suicidality	Inspired by Paris (2007) who described that, among chronically suicidal individuals, suicide ideation can function to cope with difficulties in life
7	Today I felt empty inside.	Likert	Chronic Suicidality	Item # 58 from the Borderline Symptom Inventory (Bohus et al., 2001). Feelings of emptiness are typical among chronically suicidal individuals (Paris, 2007)
8	Today I felt psychological pain (not bodily, but mental pain).	Likert	Suicidal Status Form (SSF)	Item # 1 from the Suicidal Status Form (SSF) (Jobes, 2006), translated by Gekle and Michel (personal communication, February 13, 2008) with shortened explanations of “psychic pain”
9	Today I felt stress (pressure, overwhelmed).	Likert	SSF	Item # 2 from the SSF with shortened explanations of “stress”
10	Today I was agitated (emotional urgency, restlessness, need to take action).	Likert	SSF	Item # 3 from the SSF with shortened explanations of “agitated”
11	^∗^Today I was full of hope.	Likert	SSF	Item # 4 from the SSF but with positive meaning
12	Today I felt hate or anger toward myself.	Likert	SSF	# 5 from the SSF but also including anger and without additional explanations
13	Today I felt hate or anger toward others.	Likert	SSF	To also include other-directed aggression
14	Today my suicide risk was	Visual-analog	SSF	Item # 6 from the SSF
15	Today my wish to live was	Visual-analog	SSF	Constructed by our research group, adapted from the item *I wish to live to the following extent* from the SSF
16	Today my wish to die was	Visual-analog	SSF	Constructed by our research group, adapted from the item *I wish to die to the following extent* from the SSF
17	Today I felt sad.	Visual-analog	Dysphoric Emotions	Item # 26 from the Therapy Process Questionnaire (TPQ), Haken and Schiepek (2010)
18	Today I felt guilty.	Visual-analog	Dysphoric Emotions	Item # 28 from the TPQ
19	Today I felt fear.	Visual-analog	Dysphoric Emotions	Item # 29 from the TPQ
20	^∗^My self-esteem today was	Visual-analog	Dysphoric Emotions	Item # 30 from the TPQ
21	^∗^Today I felt joy.	Visual-analog	Dysphoric Emotions	Item # 31 from the TPQ
22	Today I felt shame.	Visual-analog	Dysphoric Emotions	Item # 32 from the TPQ
23	^∗^Today I felt empathy.	Visual-analog	Dysphoric Emotions	Item # 34 from the TPQ
24	^∗^Today I thought a lot about myself.	Visual-analog	Dysphoric Emotions	Item # 35 from the TPQ
25	Today I felt insecure.	Visual-analog	Dysphoric Emotions	Item # 36 from the TPQ
26	How many drinks did you have today (0.125 liters of wine, 0.33 l of beer, 0.04 l schnapps/hard drinks, 0.08 l liqueur)	Likert	Alcohol	Constructed by our research group. The units in brackets are common in Austria
27	^∗^Last night the quality of my sleep was	Likert	Sleep quality	Constructed by our research group
28	Today I wished there was a trusted person with whom I can talk about all my personal issues.	Likert	Social support	Item # 12, emotional support subscale of the Perceived Social Support Questionnaire (Fydrich et al., 1999)
29	^∗^Today I felt like an outsider.	Likert	Social support	Item # 24, social integration subscale of the Perceived Social Support Questionnaire (Fydrich et al., 1999)
30	^∗^Today I had the impression that significant others want to decide for me what I should think and do.	Likert	Social support	Item # 54, social pressure subscale of the Perceived Social Support Questionnaire (Fydrich et al., 1999)
31	^∗^Today I wished I would receive more appreciation and affection from others.	Likert	Social support	Item # 30 satisfaction with social support subscale of the Perceived Social Support Questionnaire (Fydrich et al., 1999)
32	Today I was in touch with nature.	Likert	Contact with nature, activity, crowding	Developed by Sturm et al. (2012)
33	Today I felt that my environment was too crowded (not enough space or too many people).	Likert	Contact with nature, activity, crowding	Developed by Sturm et al. (2012)
34	Today, because of the temperature, I felt (cold/warm).	Visual-analog	Contact with nature, activity, crowding	Developed by Sturm et al. (2012)
35	Today, my mean physical activities lasted (in minutes)	Likert	Contact with nature, activity, crowding	Developed by Sturm et al. (2012)
36	Today, my intense physical activities lasted (in minutes)	Likert	Contact with nature, activity, crowding	Developed by Sturm et al. (2012)
37	Today I felt that I am part of a spiritual force in which I can trust.	Likert	Spirituality	Constructed by our research group, inspired by the item # 3 from the Self-Transcendence Scale of the Temperament and Character Inventory (Cloninger et al., 1993)
38	Today I felt a strong spiritual or emotional connection with all the people around me.	Likert	Spirituality	Item # 2 from the Self-Transcendence Scale of the Temperament and Character Inventory (Cloninger et al., 1993).

*The items were, if necessary, rephrased to focus on the present day (“today”). Likert-type response options ranged from 0 (“not at all”) to 7 (“very much”). The visual-analog scales ranged from 0 (very low/not at all/very bad) to 100 (very high/very strong/very good). ^∗^To be reversed for calculating the factor score.*

We selected two items (1, 2, numbers correspond to the items numbers in **Table 1**) from the Test of Existential Motivation (Eckhardt, 2001) which represent a construct called basic worthiness of one’s existence (in German referred to as *Grundwert*). The construct is related to the framework of existential psychology and should be strongly associated with risk for suicide (Längle, 2004).

The constructs “failed belongingness” (item 3), “perceived burdensomeness” (item 4) and “acquired capability” (item 5) (Van Orden et al., 2008) from Thomas Joiner’s Interpersonal Psychological Theory of Suicide (Joiner, 2005) were assessed by items that had the strongest item-scale correlation in a corresponding questionnaire (Van Orden, personal communication, April 12, 2009).

Item 6 represents the psychological “soothing” function of suicide ideations (Maltsberger et al., 2010) combined with the function of getting control over difficulties in life (Paris, 2007). This is characteristic of chronic suicidality, as is a strong feeling of emptiness (Paris, 2007), which was assessed by the corresponding item 7 from the Borderline Inventory (Bohus et al., 2001). Furthermore, feelings of emptiness are important to identify suicide attempters and are often present immediately preceding suicide attempts (Blasco-Fontecilla et al., 2013).

The Suicidal Status Form (Jobes, 2006) covers important suicide-related risk factors and protective factors. We used items of the Suicidal Status Form (translated by Gekle and Michel, personal communication, February 13, 2008) to assess “psychache” (item 8), “pressure” (item 9), “perturbation” (item 10), “hopelessness” (in order to avoid too many items with a negative valence this item was inverted into “hope,” item 11), “wish to live” (item 15), “wish to die” (item 16), and “risk for suicide” (item 14). The emotion of anger was divided into two aspects: “anger/hate toward others” (item 12) and “anger/hate toward self” (item 13).

Various emotions and emotion-like qualities were assessed by items taken from the “Dysphoric Emotions” subscale of the Therapy Process Questionnaire (Haken and Schiepek, 2010; Schiepek et al., 2011a): “grief” (item 17), “guilt” (item 18), “anxiety” (item 19), “self-esteem” (item 20), “joy” (item 21), “shame” (item 22), “empathy” (item 23), “self-relatedness” (item 24), and “feeling insecure” (item 25). The rationale for choosing these emotion-related items is the association of suicide risk with the experience of strong negative emotions (Hendin et al., 2010).

Daily alcohol consumption rates (item 26) and quality of sleep (item 27) are represented by one item each. Social support is assessed by four items from the German Perceived Social Support Questionnaire (Fragebogen zur sozialen Unterstützung) (Fydrich et al., 1999). From each of the four subscales, the item with the strongest item-scale-correlation was taken: “emotional support” (item 28), “social integration” (item 29), “social pressure” (item 30), and “satisfaction with social support” (item 31).

Physical activity and feeling in touch with nature was represented by five items (items 32–36) that were developed in the context of a different research purpose (Sturm et al., 2012). These items cover the known association of physical activity and depression/suicidality.

Two items assessed spiritual/religious connectedness. Item 37 was taken from the Character and Temperament Inventory (Cloninger et al., 1993). Item 38 was inspired by several items from the same inventory.

### Procedure

Currently, patients are selected to participate in the SNS monitoring if (i) they are judged to be at high risk for suicide by the team of our Suicide Prevention Department, (ii) the SNS seems to be a suitable clinical tool for this specific case at hand, and (iii) personal and temporal resources are available. Written informed consent was obtained from all participants, including the explicit statement that the SNS cannot be used to communicate with therapists. Participants complete the electronic questionnaire once per day in the evening. The monitoring is scheduled for about 100 days, resulting in a monitoring period of about three to four months (90–120 measurement points). Usually, the monitoring is extended beyond discharge from the hospital (the mean length of inpatient treatment is two to three weeks). In case participants have no internet access at home, they can borrow a laptop computer with web access. In the feedback sessions, which take place in intervals of seven to fourteen days, the time series including the results from the time series analysis are discussed with the patient and, if possible, also with the psychotherapist responsible for treatment in an outpatient setting. In these sessions, we look for specific features of the dynamics (like maxima, minima, trends, jumps, fluctuations), and ask for interpretations of these phenomena. It is intended to identify specific patterns or events that may be important to the patient. We try to focus on positive aspects like phases of successful activation of resources and competencies, or successful coping with problems. The application of the SNS in clinical practice and research has been approved by the Ethics Commission of the Salzburg Government.

## Results

The results demonstrate how a suicidal process can be monitored by the algorithms available through the SNS and interpreted from a non-linear dynamic perspective. The process description includes qualitative information from a clinician’s perspective as well as non-linear features calculated by the SNS. The clinical perspective was gained from the patient’s reports in the therapeutic sessions, by collecting daily comments, and from the feedback sessions, where time series were discussed together with the patient.

**Figure 1** depicts the time series of six selected items representing different suicide-related risk factors (raw data, **Figure 1A**) together with their related dynamic complexity (**Figure 1B**) and recurrence plots (**Figure 1C**). Important clinical information about the process is depicted in **Figure 2A**; phases of significant dynamic complexity of the item about suicide risk are given in **Figure 2B**, and phases of critical instability across items that indicate phase transitions are depicted in **Figure 2C**.

There are two phases of critical instabilities across items, as depicted in **Figure 2C**. The first critical instability across items occurred at the beginning of the monitoring process and corresponded to unusually strong fluctuations in items assessing suicide risk, hopelessness, wish to die, and experiencing pressure. Subjectively, this was a phase where the patient felt being trapped: on the one hand, she was afraid of regressing into a complete loss of control and autonomy (the awareness of being a psychiatric inpatient enhanced this fear); on the other hand, she felt incapable of living at home and was in need of help from others. Staff members in charge repeatedly discussed referring the patient to the closed ward during this period. The psychotherapist of the team interpreted this as activation of an autonomy-depencency-conflict, in line with the control-mastery theory (Silberschatz, 2005). A referral to the closed ward would have been experienced as the loss of autonomy the patient was so afraid of; however, the patient had already attempted suicide during a previous inpatient treatment (which she regretted), so that her psychiatrist was alarmed and did not want to take this risk. In addition, as was revealed later on in the psychotherapeutic process, the patient was experiencing a deep and, at this time, unconscious wish of being completely cared for. By openly addressing this issue together with the patient, she was able to understand the dilemma, to take responsibility about articulating her risk, and to protect herself against attempting suicide, thus regaining autonomy. It was necessary for the patient and the clinicians to learn that certain decisions reactivated her autonomy-dependency-conflict. These decisions concerned the change of medication, the introduction of some treatment elements, the transfer to a day clinic treatment setting, and the planning of an outpatient therapy. It was possible to delay those decisions or to allow enough time, which gave the patient feelings of autonomy and helpful support. The resolution of this apparent autonomy-dependency conflict with the clinicians may have contributed to a transition into a more stable phase with lower levels of risk and fewer fluctuations across most items.

A second critical instability across items occurred in the midst of the process between the 30th and 40th day of monitoring. Accordingly, there was an intensified period of fluctuations at different items (e.g., subjective suicide risk). From a clinical viewpoint, there was a rapid cycling of mood between days and within days. Therapy was perceived as helpful. However, the patient worried about a relaxation session she took part in with an external therapist in the first week of monitoring. The relaxation experience triggered her fear of regression into a fully mental breakdown (see the high level of “pressure” during these days) which was characterized by intense delusional fear. This worsened the patient’s situation and activated a loop of self-fulfilling prophecies. The patient experienced the strong feeling that a “crash” would be unavoidable (as had been the case in previous similar situations). Against her expectations, this crisis was able to be managed in the following therapeutic sessions. Phases of severe ruminations and hopelessness alternated with phases of hope and joyful social contacts. The critical instability preceded a change into a less emotionally charged period around the 50th day: the wish to die disappeared and perturbation was reduced; the patient’s discharge was planned in order to enable the reentrance to her workplace. This was subjectively experienced as successful, as the peaks of hope at the right side of the related panel in **Figure 1** indicate.

Before the suicide attempt, from a clinical viewpoint, sleeping difficulties reappeared with the resulting lack of energy; work seemed to be overwhelming at this time, causing a loss of hope. On day 87, the patient decided to attempt suicide but realized the same day that this was her husband’s birthday and so she changed her plans. In the time series this is visible as a sharp peak of suicide risk, wish to die, perturbation, psychache, sleep problems the night before, and several other suicide-related risk factors. The next 4 days the patient was able to delay her suicide plan, most likely because of good social contacts. It was not easy for the patient to overcome this period due to cognitive restriction. She then relapsed into the “suicidal mode,” including sleep problems, severe ruminations that centered on being dependent on her husband (visible as peaks of burdensomeness and feelings of emptiness), and a feeling of not being able to manage the future (work, partnership). In the time series this can be seen as an increased wish to die the experience of pressure and stress as well as maximum scores in the perturbation item. There was no phase of critical instability across items preceding the suicide attempt. However, in the Complexity Resonance Diagram (**Figure 2C**) there appeared to be critical dynamic complexity before the suicide attempt for some items (# 6, 9, 17, 23, 25, 27, 32, and 35). The suicide attempt was carried out on day 91 at night, with benzodiazepines, resulting in a 1-day referral to the emergency department and then to the closed ward of the psychiatric hospital.

## Discussion

The aim of this paper was to demonstrate the assessment and non-linear dynamic analysis of a suicidal process by the use of the SNS with a patient at high risk for suicide and the consequent implications for suicide research and suicide prevention. We are aware that the non-linear dynamic interpretations of the results in this paper based on a single case may seem speculative. However, our goal was not to prove but to demonstrate a concept and to outline implications for future research and clinical applications.

Out of the different non-linear qualities, we focused on critical instabilities. From a non-linear dynamic background, critical instabilities indicate phase transitions where a system is in an instable state and might move from one phase (attractor) to another one. From a psychological viewpoint, this can be interpreted as changes in cognitive/emotional/behavioral patterns or states of minds (Haken and Schiepek, 2010). The two critical instabilities across items in the time series may be interpreted as transitions into other states of mind, as inferred from the clinical impression described above (regaining autonomy, establishing hope).

From a non-linear dynamic systems perspective, suicidal states can also be viewed as specific attractors – the “suicidal mode” – characterized by severe restrictions of cognitive, emotional, and behavioral adaptivity (Rudd, 2000). Thus, before the suicide attempt, we would have expected critical instability, too, but we did not observe this across items. However, there was increased dynamic complexity for several items before the suicide attempt. Perhaps there are only specific items indicating order transitions into a suicidal mode or they may vary between individuals and some may not have been covered by our questionnaire. Thus, a prerequisite for future studies on the complexity of the suicidal process is the development of psychometrically sound research instruments that assess the components of the suicide mode. For the Salzburg Suicide Process Questionnaire, we selected items with known psychometric properties or included existing short instruments, such as the Suicidal Status Form (Jobes, 2006). However, studies are needed that investigate reliability and validity over time in high-frequent assessments. Procedures of model and item selection will contribute to the optimization of construct and predictive validity.

In addition to the application of psychometrically sound standard instruments, idiographic approaches have to be tested, i.e., by developing individualized questionnaires for each patient (Fartacek et al., 2015). This seems to be a promising development since suicide risk factors may be specific for individuals and may vary within individuals across time (Rudd, 2000). Initial experience in our department indicates that the idiographic approach may be more efficient in predicting suicide risk than standard questionnaires (Fartacek et al., 2015). As demonstrated in this case report, only certain items produced critical dynamic features before the suicide attempt. An idiographic assessment could contribute to the identification of such items and to solving the problem of inter-individual variety of suicide risk factors and warning signs (Haynes et al., 2009).

The optimal sampling rate is another important issue, as one reviewer of this paper has pointed out. In some suicide-related monitoring studies there were several (2–6) assessments per day (Links et al., 2007; Nock et al., 2009; Palmier-Claus et al., 2012, 2013; Husky et al., 2014; Law et al., 2015). Several other relevant studies used a daily sampling scheme (Clum and Curtin, 1993; Witte et al., 2006; Thompson et al., 2014; Restifo et al., 2015). We used a daily sampling scheme because this has been successfully established in the psychotherapeutic application of the SNS (Schiepek et al., 2015). Furthermore, we wanted to monitor the weeks after discharge, which are known to be a period of increased risk for suicide (Olfson et al., 2014) and we suspected that sampling more frequently than daily would be too demanding on the patients. However, studies are necessary to find out the optimal sampling rate.

Fulfilling these methodological requirements is a necessary step to further investigate the theory that suicidal processes are produced by non-linear dynamic systems. The implications from non-linear dynamic systems theory for suicidology would be crucial. Accordingly, acute suicidal crises are specific attractors. The prediction horizon is limited given that non-linear dynamic systems show complex behaviors like chaos (Schiepek et al., 2011b). This might explain the failure of long-term predictions of suicidal behavior (Large and Ryan, 2014). However, non-linear dynamic systems theory has successfully been applied in short-term prediction of problematic events in other fields of research. For example, in geophysics, methods were developed for the short-term prediction of earthquakes or tsunamis, which are based on a continuous monitoring of appropriate signals and the identification of non-linear precursors of such extreme events (Albeverio et al., 2006). This might be a promising approach for suicide research as well. If suicides or suicide attempts are preceded by a suicidal mode it would be important to know if and when people are entering the “basin” of this attractor. Critical fluctuations as described in the clinical case above can be expected before the transition into suicidal modes and may take the role of a precursor. Such critical fluctuations can be empirically assessed and their predictive ability can be statistically analyzed in future studies. Such critical fluctuations are not specific indicators of suicidal crisis. Critical fluctuations hint at phases where a system is instable and where slightest external or internal input determines the next attractor of the system, which could be a suicidal mode. Obviously, it is clinically relevant if it can be determined when a system is in such an instable phase. This goes beyond current models which try to predict suicidal behavior for time spans up to years and are thus discussed to be insufficient (Rudd et al., 2006). A continuous monitoring might improve the short-term prediction of suicidal crises and reveal phase transition-like dynamics preceding a suicidal mode. Future studies are needed that investigate the sensitivity and specifity of critical fluctuations as indicators for acute suicidal states or suicidal behavior.

One reviewer pointed out that in our present case, the increase of absolute values might be sufficient to indicate the activation of a suicidal mode, making the use of critical fluctuations as precursors obsolete. Thus, the item values itself can indicate phases of increased risk, in accordance with the warning-signs approach (Rudd et al., 2006). However, according to non-linear dynamic theory, we would expect critical fluctuations before the transition into the suicidal mode, making it a true precursor. This hypothesis needs to be studied empirically.

If non-linear dynamic process qualities are predictive of suicidal behavior, then it would be plausible to develop an early warning system based on data collected via real-time monitoring. One possibility would be to alert a professional risk manager if a defined threshold is reached. Patients may also be alerted, for example, with automatically generated e-mail or text messages that could also function as reminders of safety plans (Stanley and Brown, 2012). Accordingly, first tele-health projects have been implemented with promising results (Kasckow et al., 2015). This calls for a cooperative multi-center study in the development of early suicide warning systems.

Independent of a non-linear dynamic perspective, a fine-meshed real-time monitoring would likely improve the data quality in suicidology: shortened time lags between event and assessment reduce memory biases, ecological validity is improved by the assessment in the natural settings of an individual, and temporal trajectories of variables and their interactions can be measured (Heron and Smyth, 2010). Initial related studies have questioned the impact of established risk factors as hopelessness, which proved not to be time-correlated with suicide ideations (Ben-Zeev et al., 2012). Furthermore, affective variability may be a better predictor of suicide ideations than the average level of emotions (Palmier-Claus et al., 2012). Future research may comprise the monitoring of warning signs in high-risk individuals (Glenn and Nock, 2014), testing suicide theories longitudinally (Barzilay and Apter, 2014), or testing theories of suicide within or across patients, for example by looking at how competing risk factors from different models are associated with suicide risk (Plöderl et al., 2014).

The clinical utility and feasibility of using SNS is another topic for further research. The application of the SNS was realized with a patient at high risk for suicide. Her commitment to the monitoring procedure was high: she completed 36 items of a process questionnaire during 90 days, with only 2 days missing due to disconnected web access. This impressively high compliance has occurred in most patients using the SNS at our suicide prevention department. The regularity and quality of the feedback sessions seem to be important factors in patient compliance. The patient especially stressed the fact that the SNS did in no way increase her suicide risk. This is in line with studies not reporting harmful effects of monitoring suicide-related variables in high-risk patients (Clum and Curtin, 1993; Husky et al., 2014; Law et al., 2015). Instead, the patient experienced the daily completion of the questionnaire as a helpful routine, sometimes as an obligation, sometimes as a pastime, and sometimes it was difficult to find the appropriate answers.

It is worrisome that the patient attempted suicide despite the daily monitoring procedure, a safety plan, and a good therapeutic relationship. This is an exceptional case, but of course the question needs to be raised why this could happen despite all the efforts on behalf of the patient. According to this patient, the connection to her therapist wasn’t in her mind anymore when she was in the suicidal mode. Afterward, this was discussed at length with the patient and her husband and led to a more constructive management of future suicidal crises. In the therapeutic process after the monitoring it was helpful to be able to look back at the detailed documentation of the time before the suicide attempt.

The patient appreciated her involvement in the SNS as a tool for the improvement of her self-understanding and for a detailed process documentation that enhanced the quality of the therapy sessions. The patient had a tendency to think about herself on an abstract and explanatory level with past experiences dominating the perception of current events. The notes in the electronic diary helped her achieve a more descriptive level of self-perception and getting attached to the here and now of reality. An orientation toward more realistic thinking together with the available time-series data enhanced the quality of therapeutic behavior analysis and behavior modification procedures. An examination of the fluctuating dynamics reduced the patient’s tendency to overgeneralize negative feelings and helped her to see that social contacts effectively reduce despair. For the therapist, the data delivered by the SNS improved the reconstruction and understanding of the intersession process, similar to using diary cards (Linehan, 1993).

These positive effects are in agreement with psychotherapeutic literature suggesting that process feedback has beneficial effects in psychotherapy (Harmon et al., 2005). Besides providing feedback to the clinician that can support the early identification of non-responders and enables modification of treatment in real time (Harmon et al., 2005), it is likely that the feedback to the client herself as well as the joint reflections on the process also contribute to improvement (Harmon et al., 2005). Data from monitoring could be useful in helping accurately reconstruct the intersession process in detail with reduced recall bias (e.g., enhanced recall of information that is congruent with one’s mood and vice versa) (Wenze and Miller, 2010). This might reveal forgotten positive experiences that convey a sense of hope (Wenze and Miller, 2010). Moreover, the data may give insights into activities that may benefit patients’ emotional well-being and thus support an experience of self-efficacy and encourage self-management skills (Wichers et al., 2011) that might lead to a reduction in hopelessness (Wenzel and Beck, 2008). Besides, process feedback may draw attention to environmental contingencies and triggers (Wenze and Miller, 2010). This might improve the quality of therapeutic behavior analysis, behavior modification, and the patient’s episodic memory for triggers or warning signs (Linehan, 1993; Lynch et al., 2006). Repeated self-assessments may also increase emotional awareness and competencies in meta-cognition, as well as the awareness of suicide-specific warning signs. This is concordant with studies that found a positive association between continuous monitoring and aspects of emotion regulation (Kauer et al., 2012; Reid et al., 2013; Patzig and Schiepek, 2015).

The positive clinical experiences with SNS in our department is only anecdotal evidence, and the evidence of benefit of monitoring and feedback are plausible but mainly stem from pertinent psychotherapeutic literature. Future rigorous studies should investigate the likely beneficial and unlikely iatrogenic effects of monitoring patients at risk for suicide and the underlying mechanisms that we discussed.

## Conclusion

The real-time monitoring of suicidal processes combined with analysis tools from non-linear dynamics is technically possible. This approach is likely promising for advancing scientific developments and clinical applications in suicidology.

## Author Contributions

All authors listed, have made substantial, direct and intellectual contribution to the work, and approved it for publication.

## Conflict of Interest Statement

The authors declare that the research was conducted in the absence of any commercial or financial relationships that could be construed as a potential conflict of interest.
